# Knockdown of circEXOC6 inhibits cell progression and glycolysis by sponging miR-433-3p and mediating FZD6 in glioma

**DOI:** 10.1515/tnsci-2022-0294

**Published:** 2023-08-02

**Authors:** Yu Deng, Liu Xu, Yuqiang Li

**Affiliations:** Department of Pathology, Jinan People’s Hospital, No. 001 Xuehu Street, Zhangjiawa Street, Laiwu District, Jinan, Shandong, 271100, China

**Keywords:** glioma, circEXOC6, miR-433-3p, FZD6

## Abstract

**Background:**

The effect of circular RNA in many human cancers is widely studied. Nevertheless, their specific biological functions and mechanisms in glioma remain unclear.

**Methods:**

CircEXOC6, miR-433-3p, and frizzled class receptor 6 (FZD6) mRNA expression levels were measured by quantitative reverse transcription polymerase chain reaction assay. Cell proliferation, migration, invasion, apoptosis, and angiogenesis were tested by colony formation, cell-light 5-ethynyl-2′-deoxyuridine, transwell, and tube formation assays, respectively. Moreover, glucose consumption and lactate production were calculated to evaluate the glycolytic metabolism using the respective kits. Western blot assay was carried out to measure the protein levels of apoptotic markers (Bcl-2 and Bax), glycolytic markers (HK2 and GLUT1), and FZD6. The targeted relationship of miR-433-3p and circEXOC6 or FZD6 was verified by dual-luciferase reporter or RNA immunoprecipitation assays. *In vivo*, xenograft and immunohistochemistry assay was conducted to discriminate the effect of circEXOC6.

**Results:**

CircEXOC6 and FZD6 were highly expressed, while miR-433-3p was significantly lowly expressed in glioma tissues or cells. Deficiency of circEXOC6 inhibited cell proliferation, migration, invasion, angiogenesis, and glycolysis, and triggered cell apoptosis ratio in glioma; simultaneously, it could block the growth of tumor *in vivo*. In addition, miR-433-3p was a target of circEXOC6, and downregulated miR-433-3p could partly weaken the inhibitory effect of circEXOC6 deficiency. Besides, miR-433-3p enrichment inhibited cell progression and glycolysis in glioma, and the effect was reversed by overexpression of FZD6.

**Conclusion:**

Deletion of circEXOC6 restrained cell progression and glycolysis by sponging miR-433-3p and interacting with FZD6, which might provide an underlying target for glioma treatment.

## 1 Introduction

Glioma, the central system of malignant tumors in the brain, is deemed to be the most common primary tumor [1]. As a main element of intracranial death of patients, the characteristics of glioma are rapid metastasis and proliferation, extensive angiogenesis, high incidence and mortality rate, and poor prognosis [2]. In spite of some clinical progress that has been made, such as traditional treatment for surgery, chemotherapy, and radiotherapy, however, the patients of glioma have shown an average life span of only 14.6 months [3]. Increasing research suggested that molecular biology has been widely explored in many diseases, including glioma [4,5]. Therefore, it is necessary to comprehend the potential molecular mechanism of glioma and determine its new therapeutic method and therapeutic targets.

CircRNAs (circular RNAs), originating from precursor mRNAs, are tightly closed continuous single-chain loop structures in eukaryotic cells [6,7]. Recent advances identified that the underlying mechanisms of circRNAs emerged in various human diseases, including cancers [8,9]. For instance, circFOXO3 advanced drug sensitivity and inhibited glycolysis metabolism in non-small cell lung cancer (NSCLC) [10]. Moreover, circ_0038718 was overexpressed in colon cancer (CC), and overexpression of circ_0038718 boosted the cell capacity of proliferation, migration, and invasion in CC cells by activating the Wnt/β-catenin pathway [11]. In addition, circ_0067934 was overexpressed in papillary thyroid carcinoma (PTC), and silencing of circ_0067934 inhibited the malignant progression of PTC [12]. In glioma, mounting evidence revealed that circRNAs play a vital role in progression [13,14]. Hsa_circ_0019170 with a sequence length of 357 nt, originating from EXOC6, is located at chr10:94653105-94659401. It is currently found that circEXOC6 exhibited up-regulative expression (shown in Table S3 of Lv et al.) with whole transcriptome sequencing [15], hinting that it played a vital effect in glioma. Until now, the molecular mechanisms of circEXOC6 in human diseases have not been studied, so it provides new insights into the treatment of glioma.

Accumulating evidence revealed that circRNAs, microRNA (miRNA), and mRNA form the competitive regulatory network to regulate the physiological processes of disease [16]. A large number of data suggested that miRNA plays an oncogenic or tumor suppressor effect in a variety of tumors, for example, melanoma [17], renal cell carcinoma [18], and prostate cancer [19]. Previous studies have shown that miR-433-3p played a vital function in pancreatic cancer [20], bladder cancer [21], and glioma [22,23]. However, the mechanism of circEXOC6 and miR-433-3p is yet to be elucidated.

In this research, the mechanism of circEXOC6 in glioma was determined. We confirmed that circEXOC6 was highly expressed in glioma. Silencing cicrEXOC6 could inhibit cell proliferation, migration, invasion, angiogenesis, and glycolysis, as well as promote cell apoptosis by targeting miR-433-3p to regulate FZD6. This study establishes a new molecular therapeutic foundation for the pathogenesis of glioma.

## 2 Materials and methods

### 2.1 Tissue samples

Human glioma tumor tissues were collected from 41 glioma patients (13 patients with astrocytoma, 25 patients with glioblastoma, and 3 patients with oligodendroglioma) who were undergoing resection at Jinan People’s Hospital. A total of 41 non-glioma surgical brain tissue samples were collected as normal tissues for this study. All tissue samples were collected and stored at −80°C until employed.

### 2.2 Cell culture and transfection

Normal human astrocytes (NHAs), glioma cells (T98G, LN229, U251, and A172), and endothelial cells (HCMEC/D3) were obtained from American Type Culture Collection (Rockville, MD, USA). In our experiment, cells were incubated in Dulbecco’s modified Eagle’s medium (Thermo Fisher Scientific, Waltham, MA, USA), 10% additional fetal bovine serum (FBS; Genetimes, Shanghai, China) 100 U/mL penicillin G (Invitrogen, Carlsbad, CA, USA), and 100 µg/mL streptomycin (Invitrogen). The cells were cultured in a moist environment (Thermo Fisher Scientific) at 37°C in a 5% CO_2_ atmosphere.

Short hairpin RNA (shRNA) targeting circEXOC6 (sh-circEXOC6#1 and sh-circEXOC6#2) and the negative control (sh-NC), miR-433-3p mimic (miR-433-3p)/inhibitor (in-miR-433-3p) and the corresponding homologous negative controls (miR-NC and in-miR-NC), pcDNA3.1-FZD6 vector (FZD6), and the matched negative control pcDNA3.1 (pcDNA) were provided by RiboBio Co. Ltd (Guangzhou, China). U251 and A172 cells at 90% concentration were transfected with the abovementioned oligonucleotides or controls with Lipofectamine 3000 (Thermo Fisher Scientific).

### 2.3 Quantitative reverse transcription-polymerase chain reaction (qRT-PCR)

The total RNA of glioma tissues and cells was distilled using TRIzol reagent (Thermo Fisher Scientific). The first complementary DNA was synthesized from 200 ng of RNA with a PrimeScript RT Regent Kit (Thermo Fisher Scientific). Next, the targeting gene-specific primers were amplified using the SYBR Green mix (TaKaRa, Dalian, China). The relative expression levels of circEXOC6, miR-433-3p, and FZD6 were calculated by the methods of 2^–ΔΔCt^. The expression levels of circEXOC6, EXOC6, and FZD6 were normalized to β-actin, and small nuclear RNA U6 was considered as an internal control for miR-433-3p. All primers used in our study are shown in Table 1.

**Table 1:** Primer sequences used for PCR

Name	Primers for PCR (5′-3′)	
hsa_circ_0019170 (EXOC6)	Forward	GCAGTTATGCCTTCCTGTGC
	Reverse	TACACAGACCTTTTGGCACTCAT
EXOC6	Forward	CCAAAATGGCGGAGAACAGC
	Reverse	TCTTGTGCGCATTTGGTTGG
FZD6	Forward	GGGAACGGTGGGTTAGACG
	Reverse	CTGGGTCAATTACTCGGGGG
miR-433-3p	Forward	GTATGAATCATGATGGGCTCC
	Reverse	CTCAACTGGTGTCGTGGAG
β-Actin	Forward	CTTCGCGGGCGACGAT
	Reverse	CCACATAGGAATCCTTCTGACC
U6	Forward	CTTCGGCAGCACATATACT
	Reverse	AAAATATGGAACGCTTCACG

### 2.4 RNase R and actinomycin D assay

In the RNase R assay, the total RNA of U251 and A172 cells was extracted and co-incubated with 3 U/mg RNase R (Geneseed, Guangzhou, China) at 37°C for 30 min, and the untreated RNase R was applied as a control (RNase R-). Finally, the levels of circEXOC6 and linear EXOC6 were determined using the qRT-PCR assay. For actinomycin D treatment, U251 and A172 cells and actinomycin D (2 mg/mL, Sigma-Aldrich, St. Louis, MO, USA) were co-cultured for 0, 4, 8, and 12 h at 37°C. The expression levels of circEXOC6 and linear EXOC6 were detected using qRT-PCR analysis.

### 2.5 Cell proliferation

For colony formation assay, the glioma cells of U251 and A172 were harvested and cultured in six-well plates at a thickness of 300 cells/well at 37°C for incubation. After culturing for 14 days, the forming colony cells were fixed with paraformaldehyde (4%, Beyotime, Beijing, China) for 10 min, stained with crystal violet (0.5%, Beyotime) for 30 min, and washed three times with phosphate-buffered saline (PBS). The number of visible colonies was photographed, manually recorded, and statistically analyzed.

Following transfection, U251 and A172 cells were cultured in 96-well plates at a thickness of 3 × 10^4^ cells/well and incubated for 48 h. Next, 50 µmol/L 5-ethynyl-2′-deoxyuridine (EdU) labeling solution was added to the culture of each well for 2 h. Then, the cells were fixed with a 4% fixative solution (Sigma-Aldrich) for 30 min and washed twice with PBS. Afterward, 0.5% TritonX-100 (Sigma-Aldrich) was added and stained with the EdU detection kit (RiboBio, Guangzhou, China). Finally, the green-colored positive cells and blue-colored nuclei were observed and photographed using a fluorescence microscope (Olympus, Tokyo, Japan). The proliferative ability of cells was determined by calculating the positive cell rates.

### 2.6 Wound healing assay

U251 and A172 cells were seeded in 24-well plates and gestated. The wound emerged in a beeline by scratching the cell layer with a sterile pipette tip. The cells were washed using PBS to detach the suspended cells. The wound surface was observed and imaged at 0 and 24 h under an optical microscope (Olympus).

### 2.7 Transwell assay

The migration and invasion of U251 and A172 cells were analyzed with 24-well transwell chambers (Corning, Tewksbury, MA, USA). In brief, the upper chamber was supplemented with 200 μL of serum-free medium in the migration assay, and diluted Matrigel (Bedford, MA, USA) was added in the invasion assay. In the migration and invasion assay, a 15% FBS cell medium was added to the lower chamber uniformly at 37°C in a 5% CO_2_ incubator. At 24 h after culture, the cells were fixed with 4% paraformaldehyde (Sigma-Aldrich) and dyed with 0.1% crystal violet (Sigma-Aldrich). The stained migration and invasion cells were viewed and imaged using a light microscope (magnification, ×100; Nikon, Tokyo, Japan).

### 2.8 Flow cytometry analysis

U251 and A172 cells (1 × 10^6^) were collected and re-suspended in the binding buffer with 500 µL of Annexin V. About 5 μL of Annexin V-FITC and 10 μL of propidium iodide were double-stained at 4°C in the dark. After 15 min, flow cytometry (Beckman, Miami, FL, USA) was used to monitor and analyze the apoptotic cells.

### 2.9 Tube formation assay

The 96-well plates were coated with pre-cooled matrigel (80 μL/well; BD Biosciences, Bedford, MA, USA) and incubated for 30 min at 37°C. HCMEC/D3 cells (5 × 10^4^) were added to the plate and cultured with transfected U251 and A172 cells’ medium for 24 h. After co-culture, the tube formation was photographed under a microscope, and angiogenesis was quantitated by counting the tubule length.

### 2.10 Glucose consumption and lactate production assay

A glucose assay kit (Abnova, Taiwan, China) and a lactate assay kit (KeyGen, Nanjing, China) were employed to test the levels of glucose consumption and lactate production in U251 and A172 cells. The cells (1 × 10^5^/well) were plated in 12-well plates and incubated overnight. The glucose consumption and lactate production were monitored by the corresponding kits according to the manufacturer’s instructions.

### 2.11 Western blot assay

The glioma tissues and cells (U251 and A172) were collected and the total proteins were extracted with the RIPA buffer (Beyotime). The concentration of protein was calculated with a bicinchoninic acid protein quantification kit (GLPBIO, Montclair, USA). Next, 15% sodium dodecyl sulfate-polyacrylamide gel electrophoresis was used to separate the proteins (30 μg) and transferred to a polyvinylidene fluoride membrane (PVDF) (Millipore, Billerica, MA, USA). Next, the PVDF membrane was blocked with 5% skim milk, and then the co-cultured PVDF membrane and primary antibody were incubated overnight at 4°C. The primary antibody was as follows: anti-Bcl-2 (Abcam, 1:500, ab32124), anti-Bax (Abcam, 1:500, ab182733), anti-HK2 (Abcam, 1:500, ab209847), anti-GLUT1 (Abcam, 1:500, ab150299), anti-FZD6 (Abcam, 1:500, ab98933), and anti-β-actin (Abcam, 1:500, ab7817). After washing with the TBST buffer, goat anti-rabbit IgG H&L (HRP) (Abcam, 1:20,000, ab205718) was added to the PVDF membrane and incubated for 2 h. The immunoblots were analyzed with the ECL reagent (Santa Cruz Biotechnology). In this study, β-actin level was utilized as an internal control.

### 2.12 Dual-luciferase reporter assay

The targeting relationship of miR-433-3p and circEXOC6 or FZD6 was predicted using the website of circinteractome (https://circinteractome.nia.nih.gov/) or starbase (http://starbase.sysu.edu.cn). Next, the wild-type (WT) circEXOC6 or FZD6 (circEXOC6 WT, FZD6 3′-UTR WT) or mutant (MUT) circEXOC6 or FZD6 (circEXOC6 MUT, FZD6 3′-UTR MUT) of miR-433-3p binding sites were constructed, and cloned into a pmirGLO vector (Promega Corporation). Next, the WT or MUT vectors and the miRNA mimic or miR-433-3p mimic were co-transfected in U251 and A172 cells. After 48 h, the Dual-Luciferase Reporter Assay Kit (Sangon, Shanghai, China) was employed to calculate the luciferase activity.

### 2.13 RNA immunoprecipitation (RIP) assay

Magna RIP kit (Millipore) was used to confirm the relationship between circEXOC6 and miR-433-3p. In a nutshell, U251 and A172 cells were lysed and incubated with RIP lysis buffer (Millipore) for 48 h. Magnetic beads with human Argonaute 2 (Ago2) antibody or IgG antibody were conjugated and added with cell lysates and cultured overnight at 4°C. Co-precipitated RNAs of circEXOC6 and miR-433-3p were determined by using qRT-PCR.

### 2.14 *In vivo* study

In our study, the animal test was authorized by the Animal Experimentation Ethics Committee of Jinan People’s Hospital. Six-week old BALB/c nude mice were obtained from the Shanghai Experimental Animal Center (SLAC, Shanghai, China). The mice in our study were freely divided into two groups with six in each group. A172 cells were transfected with sh-NC or sh-circEXOC6#1, which was subcutaneously injected into nude mice to structure the xenograft models. Once a week for 28 days, we measured the tumor volume. The tumor was weighed at 28 days of sacrificed mice. QRT-PCR or western blot assays were carried out to check the levels of circEXOC6, miR-433-3p, and FZD6.

In the immunohistochemistry (IHC) assay, tumors tissues were sectioned (5 μm thick) in paraffin. The primary antibody anti-FZD6 (Abcam, 1:1,000, ab150545) and the slices were co-incubated in a refrigerator overnight at 4°C after dewaxing and repair, and cultured with the secondary antibody (Abcam, 1:50,000, ab205718). After washing twice with the PBS solution, the slices were stained with diaminobenzidine for 3 min and subsequently re-dyed with hematoxylin (Beyotime). Finally, it was photographed under a light microscope (Nikon).

### 2.15 Statistical analysis

The data analysis and graphing were carried out using GraphPad Prism 8.0 (GraphPad Inc., San Diego, CA, USA). All data were presented as mean ± standard deviation of three replicates. Comparisons between the two groups were done using Student’s *t-*test. ANOVA was used to compare three or more groups. *P < 0.05 clearly indicated that the difference was statistically significant.


**Ethical approval:** The research related to human use complied with all the relevant national regulations and institutional policies, and in accordance with the tenets of the Helsinki Declaration, and has been approved by the author’s institutional review board or equivalent committee. The present study was approved by the ethical review committee of Jinan People’s Hospital.
**Informed consent:** Informed consent has been obtained from all individuals included in this study.

## 3 Results

### 3.1 CircEXOC6 expression was elevated in glioma tissues and cells

**Figure 1:**
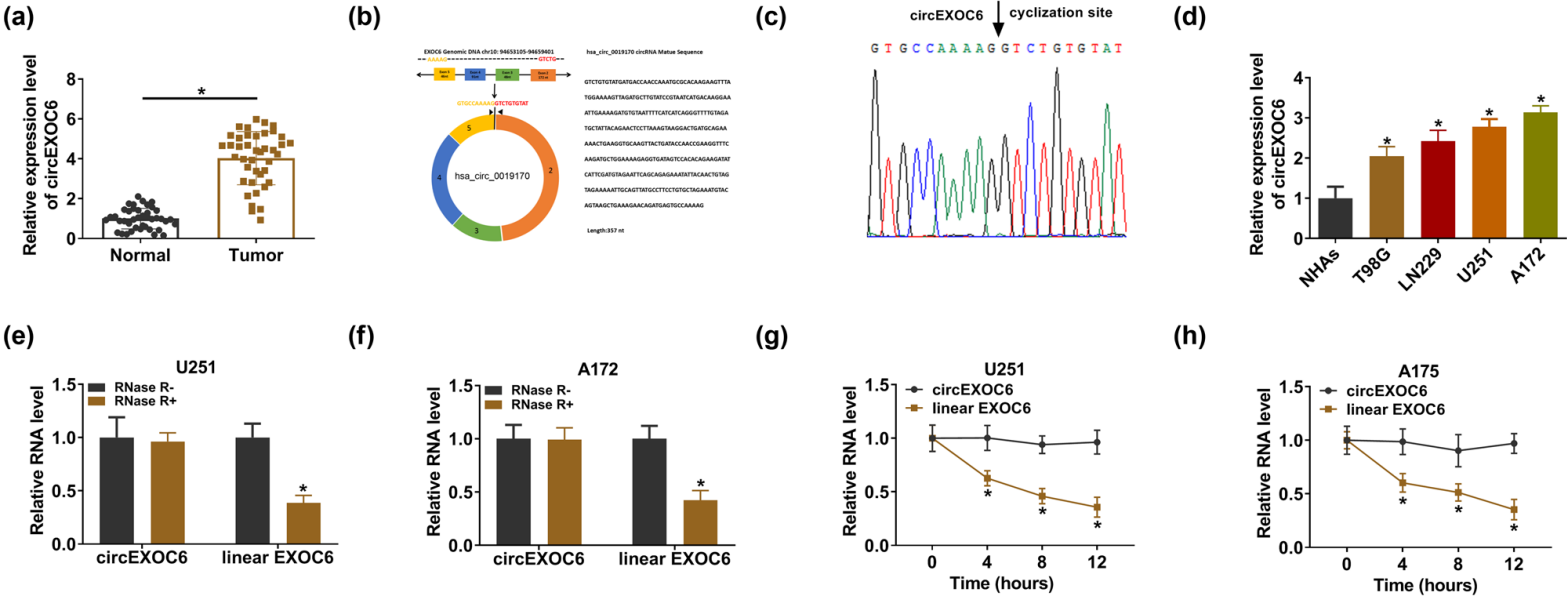
CircEXOC6 was overexpressed in glioma sample tissues and cells. (a) CircEXOC6 expression was measured by qRT-PCR in glioma tissues and normal tissues (*n* = 41). (b) The structural representation of cicrEXOC6. (c) Sanger sequencing was applied to test amplification products. (d) The expression of circEXOC6 was analyzed using qRT-PCR in glioma cells (T98G, LN229, U251, and A172) and NHAs. (e) and (f) CircEXOC6 expression was examined after RNase Rdigestion in U251 and A172 cells. (g) and (h) The half-life of circEXOC6 and linear EXOC6 after treatment with actinomycin D at the indicated time points for 0, 4, 8, or 12 h in U251 and A172 cells. All experiments were performed in triplicate, with each independent experiment set three times to generate an average value. *P < 0.05.

At the outset, the expression level of circEXOC6 was detected in 41 glioma tumor tissues and normal tissues. The results from qRT-PCR revealed that circEXOC6 was notably elevated in glioma tumor tissues with reference to normal tissues (Figure 1(a)). Besides, we also detected the expression of circEXOC6 in different types of gliomas, and the results indicated that the expression of circEXOC6 in different types of glioma was higher than in normal tissues (Figure S1(a)). CircEXOC6 (357 base pairs in length) consisted of exons 2–5 of its parental gene EXOC6 and was located on chr10:94653105-94659401 (Figure 1(b)). In addition, the amplification products were further validated by the back-splicing junction of circEXOC6 by Sanger sequencing (Figure 1(c)). As shown in Figure 1(d), qRT-PCR results confirmed that circEXOC6 was obviously upregulated in glioma cell lines (T98G, LN229, U251, and A172) compared with the normal cell line NHAs. In addition, glioma cells were treated with RNase R or actinomycin D to confirm the stability of circEXOC6. The results showed that circEXOC6 had strong tolerance to RNase R digestion, while linear EXOC6 was totally degraded in U251 and A172 cells (Figure 1(e) and (f)). Meanwhile, U251 and A172 cells were cultured with actinomycin D. The half-life of circEXOC6 was significantly longer than that of linear EXOC6. The stability of circEXOC6 was further confirmed (Figure 1(g) and (h)). To sum up, our findings reveal that the expression of circEXOC6 was upregulated in glioma tissues and cells.

### 3.2 Knockdown of circEXOC6 inhibited the proliferation, migration, invasion, angiogenesis, and glycolysis, and facilitated apoptosis of glioma cells

**Figure 2:**
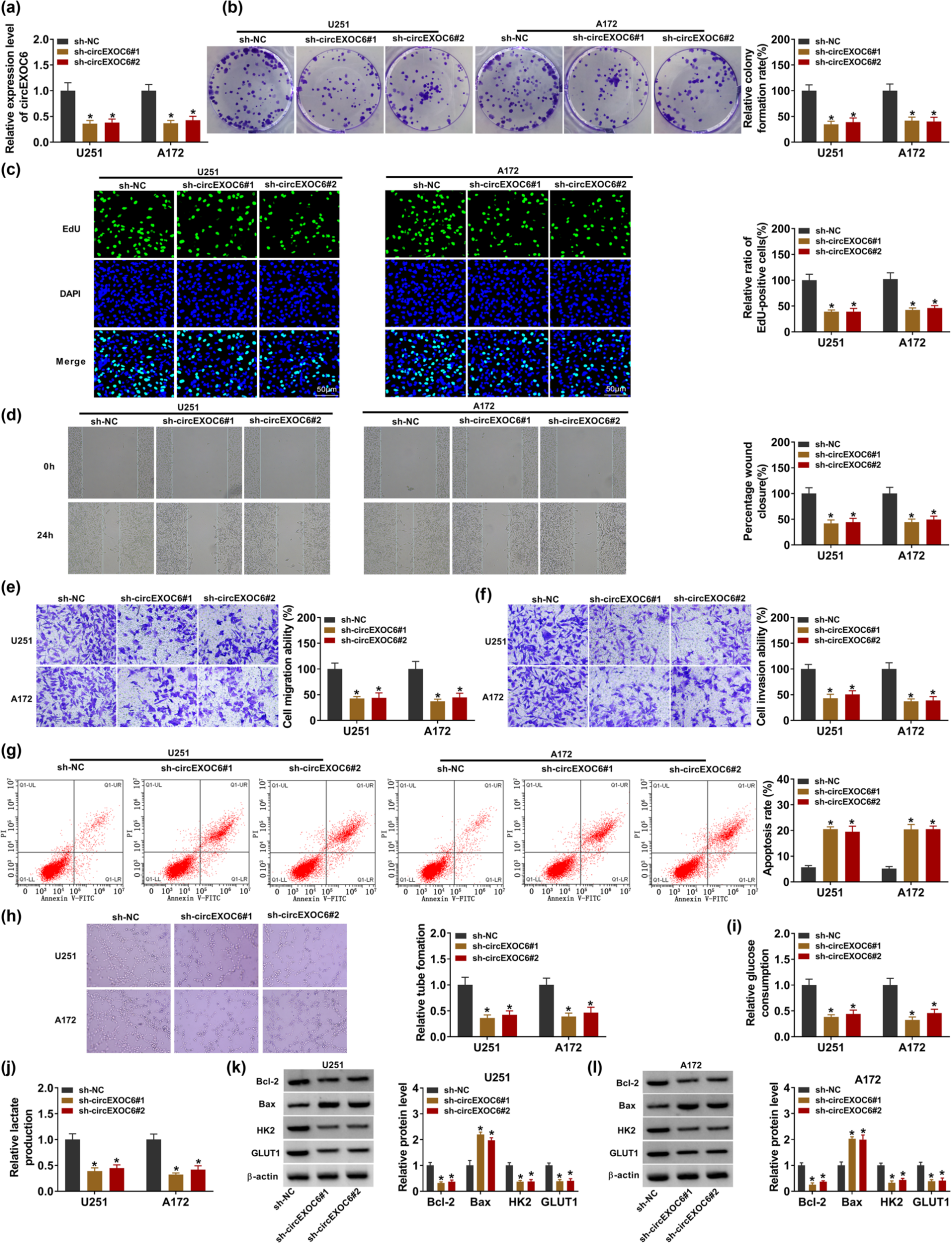
Lowly expressed circEXOC6 inhibited the proliferation, migration, invasion, angiogenesis, and glycolysis, and boosted the apoptosis of glioma cells. (a–l) U251 and A172 cells were transfected with sh-NC, sh-circEXOC6#1, or sh-circEXOC6#2. (a) The efficiency of shRNA sequences targeting circEXOC6 was analyzed in U251 and A172 cells. (b) and (c) Colony formation and EdU assay were used to check cell proliferation. (d)–(f) Wound healing and transwell assays were performed to assess cells migration and invasion. (g) Cell apoptosis was measured by flow cytometry assay. (h) Tube formation assay was used to determine tumor angiogenesis. (i) and (j) Glucose consumption and lactate production were analyzed to assess cell glycolysis. (k)–(l) The protein levels of apoptosis-related proteins (Bcl-2 and Bax) and glycolysis-related proteins (HK2 and GLUT1) were examined by western blot assay. All experiments were performed in triplicate, with each independent experiment set three times to generate an average value. *P < 0.05.

To further investigate the molecular mechanism of circEXOC6 in glioma cells, two shRNAs were designed targeting the unique back-splicing site of circEXOC6 (sh-EXOC6#1 and sh-EXOC6#2) and control (sh-NC) in U251 and A172 cells. The results from qRT-PCR revealed that the relative expression of circEXOC6 was strikingly reduced in sh-EXOC6#1 and sh-EXOC6#2 groups compared with that in sh-NC (Figure 2(a)). The cell proliferation ability was determined by colony formation and EdU assay. These data indicated that cell proliferation abilities were significantly suppressed by circEXOC6 silencing in U251 and A172 cells (Figure 2(b) and (c)). In addition, wound healing and transwell assays showed that knockdown of circEXOC6 inhibited cell migratory and invasive abilities of U251 and A172 cells (Figure 2(d)–(f)). Simultaneously, the cell apoptotic rate was increased by the deficiency of circEXOC6 in U251 and A172 cells, which was determined by flow cytometry assay (Figure 2(g)). The tube formation assay indicated that circEXOC6 knockdown confined tumor angiogenesis (Figure 2(h)). The circEXOC6 deletion could markedly weaken the levels of glucose consumption and lactate production in U251 and A172 cells (Figure 2(i) and (j)). Accordingly, the apoptosis-related proteins (Bcl-2 and Bax) and glycolysis-related proteins (HK2 and GLUT1) were shown by western blot assays. The data suggested that the protein levels of Bcl-2, HK2, and GLUT1 were decreased, while the Bax protein level was remarkably elevated in U251 and A172 cells (Figure 2(k) and (l)). All data exhibited that circEXOC6 deficiency suppressed the progression of glioma.

### 3.3 CircEXOC6 served as a sponge of miR-433-3p in glioma cells

**Figure 3:**
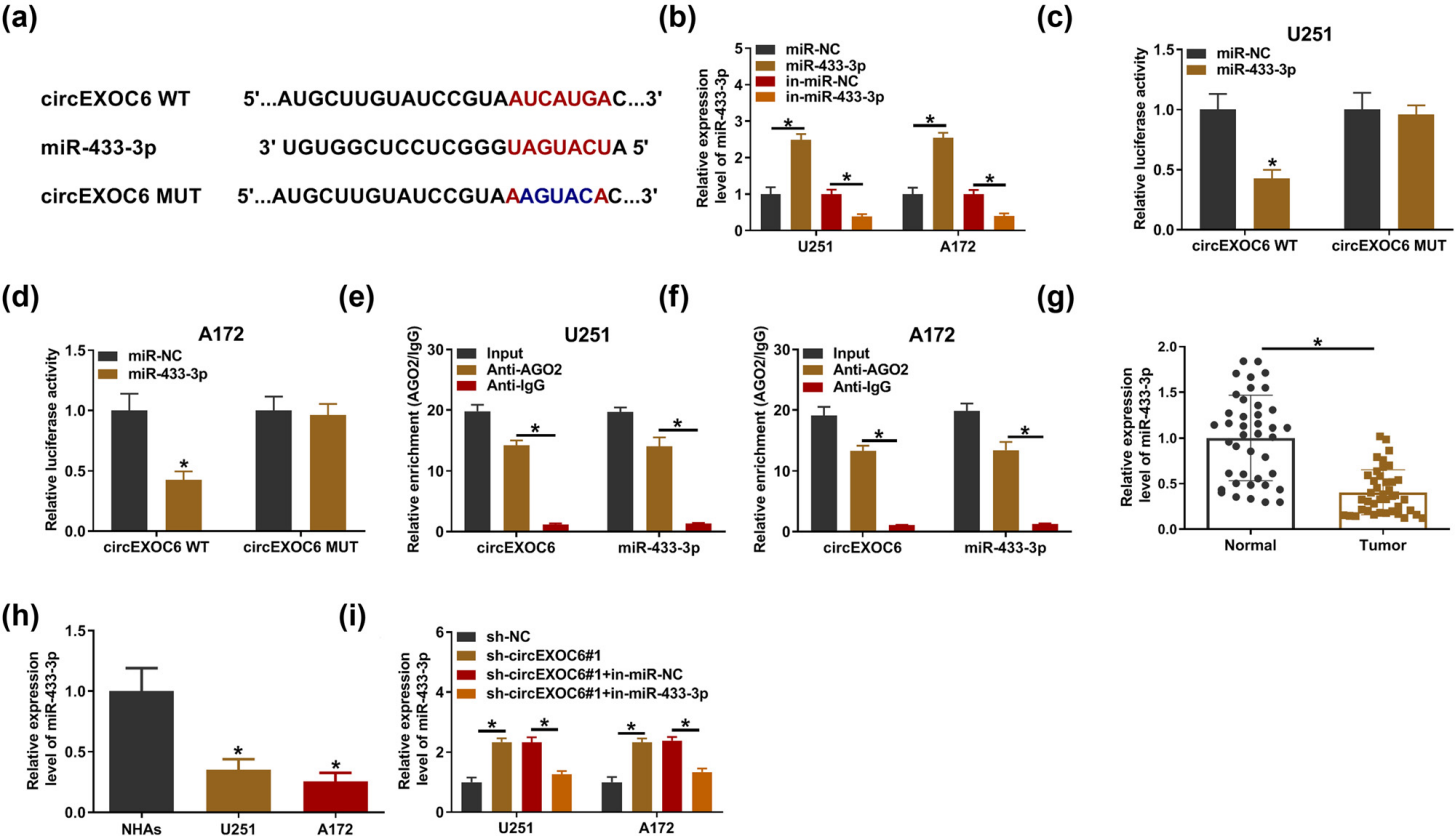
MiR-433-3p was a target of cirEXOC6. (a) The binding sites between miR-433-3p and circEXOC6 were predicted by circinteractome. (b) The up-regulation or down-regulation efficiency of miR-433-3p was examined by qRT-PCR in U251 and A172 cells. The binding relationship between circEXOC6 and miR-433-3p was verified by dual-luciferase reporter assay (c) and (d), and RIP assay (e) and (f) in U251 and A172 cells. (g) and (h) The expression of miR-433-3p in glioma tissues and cells was detected by qRT-PCR. (i) The miR-433-3p expression level was examined in U251 and A172 cells transfected with sh-NC, sh-circEXOC6#1, sh-EXOC6#1 + in-miR-NC, or sh-circEXOC6#1 + in-miR-433-3p. All experiments were performed in triplicate, with each independent experiment set three times to generate an average value. *P < 0.05.

To further illuminate the role of circEXOC6 in regulating glioma progression, the bioinformatics analysis was carried out in our study. Circinteractome (https://circinteractome.nia.nih.gov/) software illustrated that circEXOC6 had potential binding sites with miR-433-3p (Figure 3(a)). Then, miR-433-3p expression was detected in U251 and A172 cells after transfection with the miR-433-3p mimic or inhibitor, and it was found that miR-433-3p was overexpressed after transfection with the miR-433-3p mimic and it was downregulated after transfection with miR-433-3p inhibitor (Figure 3(b)). The results from the dual-luciferase reporter assay indicated that miR-433-3p overexpression visibly restrained the circEXOC6 WT reporter activity, whereas the luciferase reporters of circEXOC6 MUT were not affected by the transfected miR-433-3p mimic or miR-NC in U251 and A172 cells (Figure 3(c) and (d)). Moreover, the data from the RIP assay proved that circEXOC6 and miR-433-3p were markedly enriched in Ago2 pellets with reference to the IgG group in U251 and A172 cells (Figure 3(e) and (f)). In glioma tissues and cells, miR-433-3p was remarkably depressed (Figure 3(g) and (h)). After that, the results from qRT-PCR indicated that the knockdown of circEXOC6 elevated the expression level of miR-433-3p, while co-transfection of the miR-433-3p inhibitor could weaken this effect in U251 and A172 cells (Figure 3(i)). Thus, these results supported that the miR-433-3p expression level was negatively regulated by circEXOC6 in glioma cells.

### 3.4 miR-433-3p inhibition returned the inhibitory effects of circEXOC6 knockdown on glioma cells

**Figure 4:**
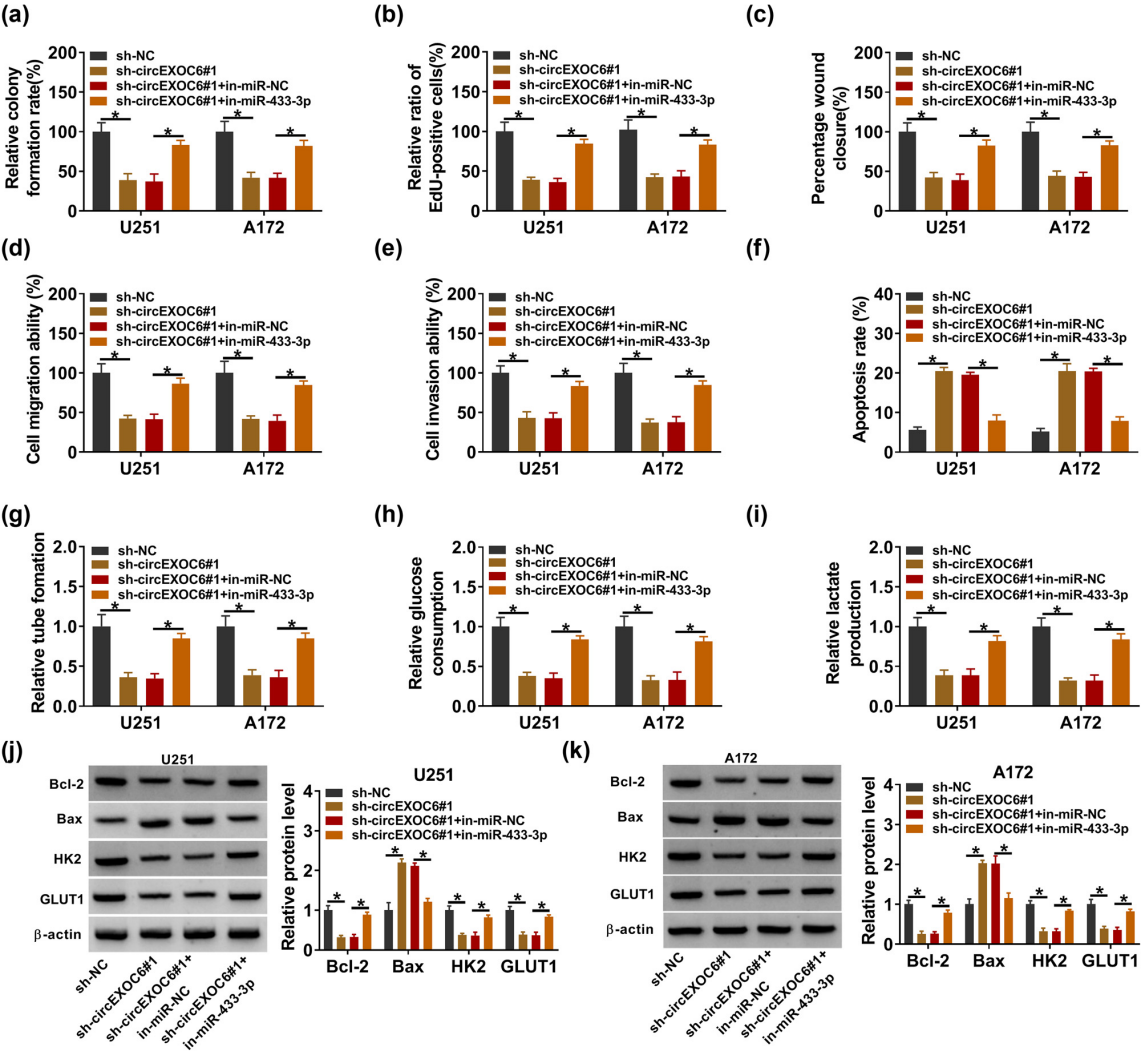
Knockdown of circEXOC6 inhibited glioma cell progression by sponging miR-524-5p. (a)–(k) U251 and A172 cells were transfected with sh-NC, sh-circEXOC6#1, sh-EXOC6#1 + in-miR-NC, or sh-circEXOC6#1 + in-miR-433-3p. (a) and (b) Cell proliferative ability was evaluated using colony formation and EdU assay. (c)–(e) Wound healing and transwell assays were performed to assess cells’ migration and invasion. (f) Cell apoptosis was analyzed by flow cytometry assay. (g) Angiogenesis was evaluated by tube formation assay. (h) and (i) Glucose consumption and lactate production were measured to assess cell glycolysis. (j) and (k) The protein levels of apoptosis-related proteins (Bcl-2 and Bax) and glycolysis-related proteins (HK2 and GLUT1) were analyzed by western blot assay. All experiments were performed in triplicate, with each independent experiment set three times to generate an average value. *P < 0.05.

The rescue experiments were carried out to investigate the mechanism of circEXO6 in regulating cell progression by miR-433-3p in glioma cells. U251 and A172 cells were transfected with sh-NC, sh-circEXOC6#1, sh-circEXOC6#1 + in-miR-NC or sh-circEXOC6#1 + in-miR-433-3p. The results from colony formation and EdU assay suggested that inhibition of miR-433-3p partially weakened the inhibitory effects of sh-circEXOC6#1 in U251 and A172 cell proliferation (Figure 4(a) and (b)). Moreover, downregulated miR-433-3p largely blocked the suppressed effect on cell migration and invasion that was induced by the absence of circEXOC6 (Figure 4(c)–(e)). Concurrently, the findings showed that the enhancing effect of circEXOC6 deficiency on the cell apoptosis ratio was reverted by the miR-433-3p inhibitor (Figure 4(f)). Besides, co-transfection of in-miR-433-3p reversed the suppressive effect on tumor angiogenesis of circEXOC6 knockdown (Figure 4(g)). Furthermore, the knockdown of circEXOC6 inhibited glucose consumption and lactate production that could be partly abrogated by co-transfection of miR-433-3p inhibition in U251 and A172 cells (Figure 4(h) and (i)). Correspondingly, downregulation of miR-433-3p could largely rescue circEXOC6 silencing mediated by the protein changes of Bcl-2, Bax, HK2, and GLUT1 (Figure 4(j) and (k)). In a word, our work exhibited that silencing of circEXOC6 inhibited the malignant phenotype of glioma via sponging miR-433-3p.

### 3.5 FZD6 was a target of miR-433-3p

**Figure 5:**
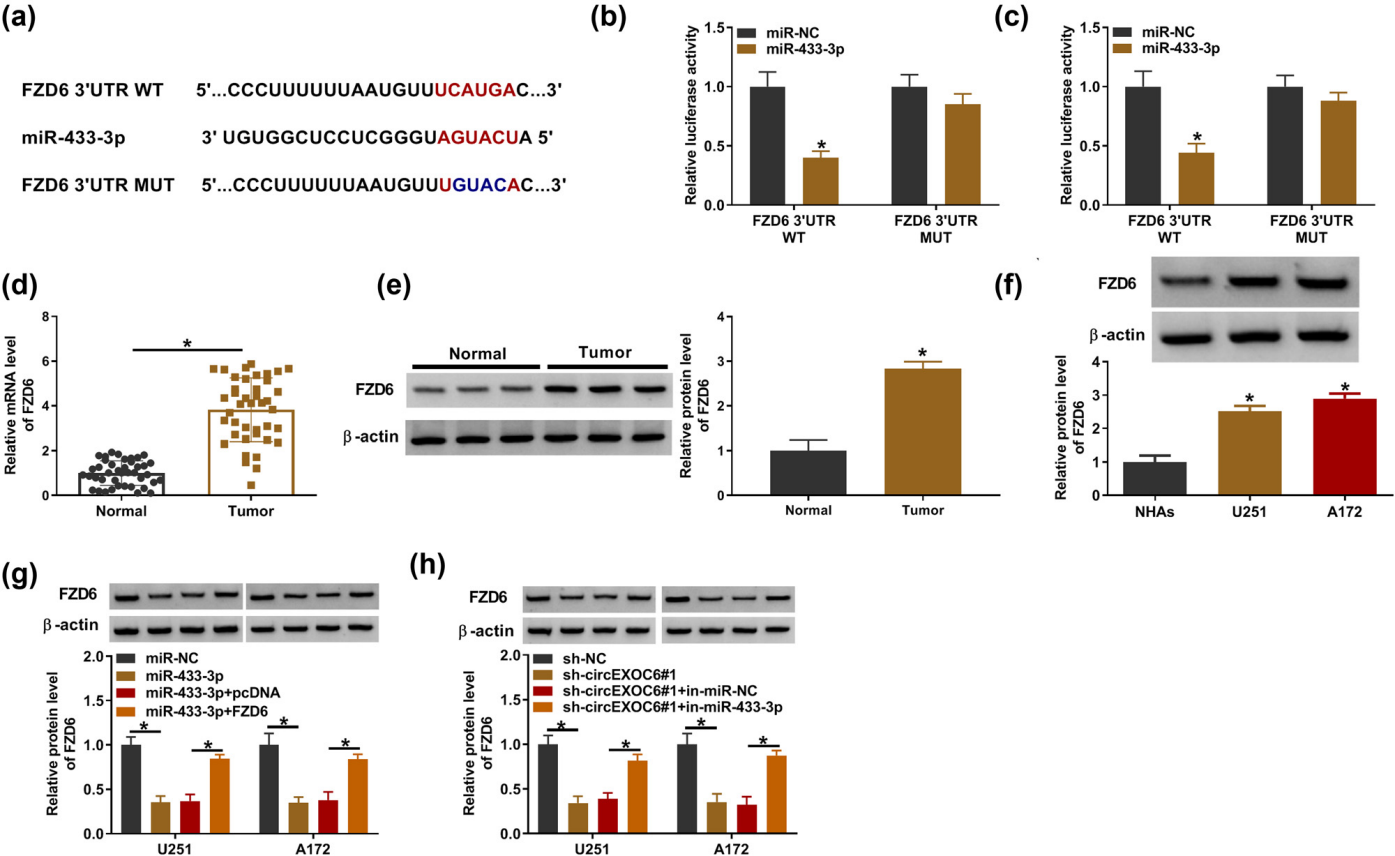
CircEXOC6 mediated FZD6 expression by targeting miR-433-3p. (a) FZD6 was predicted to be the target of miR-433-3p by starbase. (b) and (c) Dual-luciferase reporter assay was used to validate the interaction between miR-433-3p and FZD6. (d) and (e) The mRNA and protein levels of FZD6 were evaluated in glioma tissues and normal tissues. (f) The protein level of FZD6 in glioma cells was determined by western blot. (g) The protein level of FZD6 was examined in U251 and A172 cells transfected with miR-NC, miR-433-3p, miR-433-3p + pcDNA, or miR-433-3p + FZD6. (h) The protein level of FZD6 was evaluated in U251 and A172 cells transfected with sh-NC, sh-circEXOC6#1, sh-EXOC6#1 + in-miR-NC, or sh-circEXOC6#1 + in-miR-433-3p. All experiments were performed in triplicate, with each independent experiment set three times to generate an average value. *P < 0.05.

The starbase software (http://starbase.sysu.edu.cn) predicted that miR-433-3p had more targeted mRNA. Through literature research, we screened out five mRNAs (SPARC, YY1, ERBB4, MAP3K2, and FZD6) that are highly expressed in glioma tumor tissues and promote glioma progression. Then, qRT-PCR analysis showed that miR-433-3p significantly suppressed the expression of FZD6 (Figure S1(b)), so FZD6 was selected as the target of miR-433-3p for this study. The complementary binding sites between miR-433-3p and FZD6 are shown in Figure 5(a). Dual-luciferase reporter assay revealed that the luciferase activity was evidently decreased by co-transfection of miR-433-3p and FZD6 3′-UTR WT in U251 and A172 cells (Figure 5(b) and (c)). Besides, the mRNA and protein expression of FZD6 was clearly overexpressed in glioma tissues and cells with reference to normal samples and cells by qRT-PCR and western blot assay (Figure 5(d) and (e)). Meanwhile, the results showed that the protein expression level of FZD6 was strikingly upregulated in U251 and A172 cells compared with NHAs (Figure 5(f)). In addition, the elevation of miR-433-3p could restrain the FZD6 protein level, and the inhibition effect was recovered by FZD6 overexpression in U251 and A172 cells (Figure 5(g)). Furthermore, the protein level of FZD6 was inhibited by the silent circEXOC6, while co-transfected in-miR-433-3p could reverse this effect (Figure 5(h)). These findings indicated that circEXOC6 sponged miR-43-3p to regulate the expression of FZD6 in glioma cells.

### 3.6 miR-433-3p regulated glioma progression by targeting FZD6

**Figure 6:**
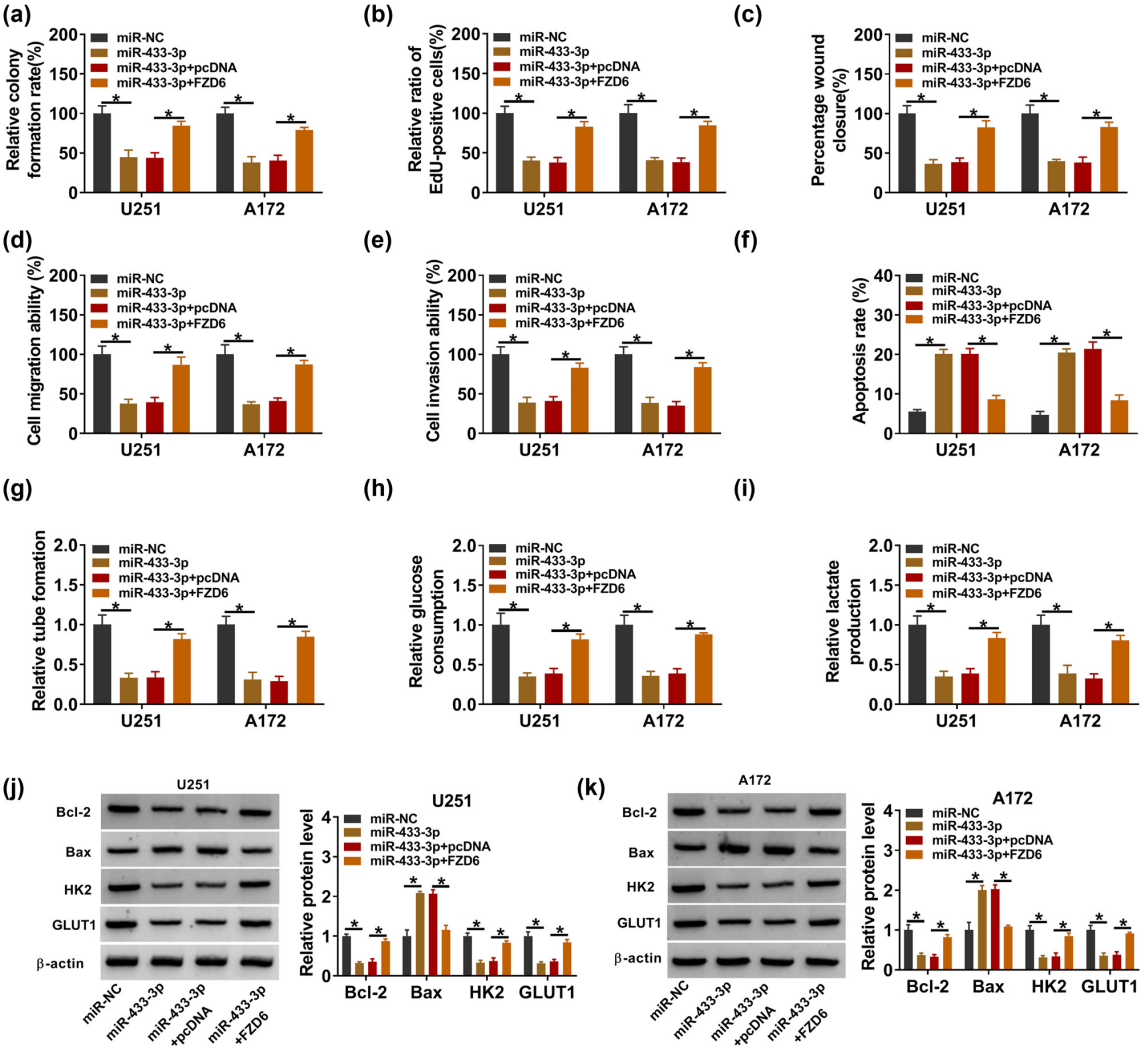
FZD6 overexpression reversed the effect of miR-433-3p mimic on glioma cell progression. (a)–(k) U251 and A172 cells were transfected with miR-NC, miR-433-3p, miR-433-3p + pcDNA, or miR-433-3p + FZD6. (a) and (b) Cell proliferative capacity was determined by colony formation and EdU assay. (c)–(e) Wound healing and transwell assays were carried out to assess cells migration and invasion. (f) Cell apoptosis was evaluated by flow cytometry assay. (g) Angiogenesis was determined using the tube formation assay. (h) and (i) Glucose consumption and lactate production were determined to assess cell glycolysis. (j) and (k) The protein levels of apoptosis-related proteins (Bcl-2 and Bax) and glycolysis-related proteins (HK2 and GLUT1) were evaluated by western blot assay. All experiments were performed in triplicate, with each independent experiment set three times to generate an average value. *P < 0.05.

To evaluate whether miR-433-3p modulated the progression of glioma cells by targeting FZD6, U251, and A172 cells were transfected with miR-NC, miR-433-3p, miR-433-3p + pcDNA, or miR-433-3p + FZD6. In this research, cell proliferation was suppressed by overexpressed miR-433-3p, and this effect was eliminated by co-transfection of FZD6 by colony formation and EdU assay (Figure 6(a) and (b)). Elevating miR-433-3p could inhibit cell migration and invasion, while the role was eliminated by overexpressed FZD6 (Figure 6(c)–(e)). At the same time, our work found that miR-433-3p mimics advanced cell apoptosis and suppressed angiogenesis, while overexpressed FZD6 could largely alleviate this effect (Figure 6(f) and (g)). Glucose consumption and lactate production were inhibited by the miR-433-3p mimic, whereas FZD6 overexpression blocked this effect (Figure 6(h) and (i)). The data from western blot assay revealed that upregulated miR-433-3p inhibited the protein levels of Bcl-2, HK2, and GLUT1 and promoted Bax protein expression; these effects were partly recovered by overexpression of FZD6 (Figure 6(j) and (k)). To sum up, all of these results revealed that miR-433-3p regulated the development of glioma cells by targeting FZD6.

### 3.7 Interference of circEXOC6 repressed the tumor growth of glioma *in vivo*

**Figure 7:**
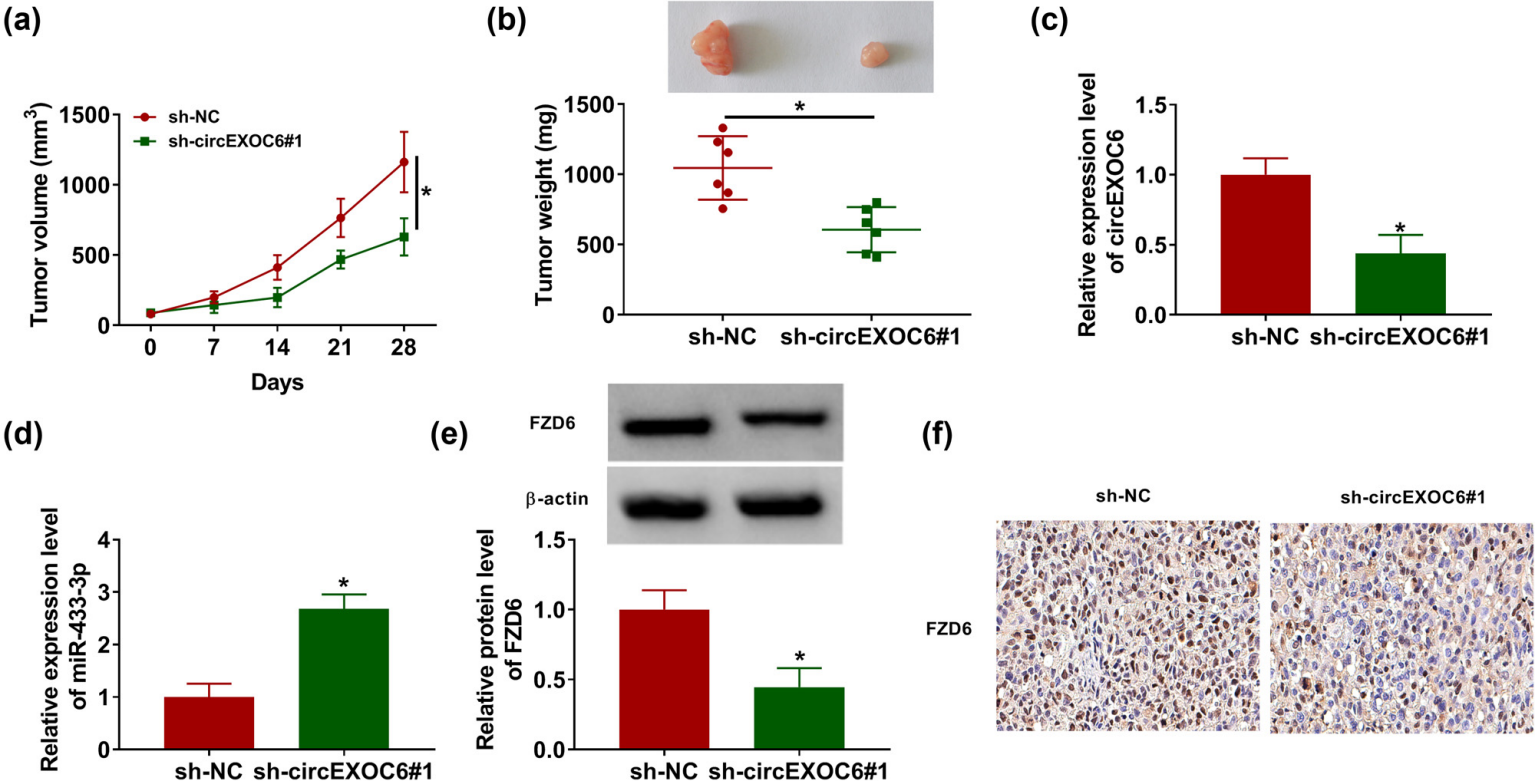
Downregulated circEXOC6 hindered glioma growth *in vivo*. A172 cells containing sh-NC or sh-circEZOC6#1 were subcutaneously injected into nude mice. (a) Tumor volumes were evaluated every one week. (b) Tumor size was measured after 28 days. (c)–(e) The expression levels of circEXOC6, miR-433-3p, and FZD6 were determined by qRT-PCR or western blot assay. (f) FZD6 expression in the xenograft tumor was determined by using IHC. All experiments were performed in triplicate, with each independent experiment set three times to generate an average value. *P < 0.05.

Finally, a xenograft glioma mouse model was constructed to determine the effect of circEXOC6. The results revealed that the volume and weight of the tumors were significantly reduced by knockdown of circEXOC6 (Figure 7(a) and (b)). Meanwhile, the data from the qRT-PCR assay indicated that circEXOC6 expression was notably decreased and the expression level of miR-330-5p was increased in the sh-circEXOC6#1 group compared with that in sh-NC (Figure 7(c) and (d)). The western blot assay indicated that the silencing of circEXOC6 remarkably restrained the protein expression of FZD6 (Figure 7(e)). Simultaneously, the findings from IHC exhibited that the number of positive cells for FZD6 was abated in circEXOC6 silencing with reference to sh-NC (Figure 7(f)). All of these assays indicated that silencing of circEXOC6 blocked the growth of tumors *in vivo*.

## 4 Discussion

As the most common malignant intracranial invasive disease, glioma is accompanied by high invasiveness and poor prognosis [24]. CircRNAs play a pivotal effect in many cancers. Hence, the results from our study suggested that the knockdown of circEXOC6 regulated FZD6 by targeting miR-433-3p to restrain the cell progression of glioma, which was hoped to provide a new perspective for glioma treatment.

CircRNAs are a kind of non-coding RNAs and lack the structure of 5′-cap and 3′-polyA tail, more stable than the linear RNA [25]. In glioma, previous research has indicated that circRNAs played an important effect in the development of glioma. For example, the circMMP1 expression level was markedly improved in glioma tissues and cells and the deficiency of circMMP1 could block the cells occurrence and development [26]. Previous studies showed that upregulated circGLIS3 advanced the ability of migration and invasion in glioma cells [27]. Furthermore, hsa_circ_0033009 (circLGMN) was related to poor prognosis in glioma patients and upregulated circLGMN significantly advanced the proliferative and invasive capacities [28]. Recently, Lv et al. found that circEXOC6 was overexpressed by transcriptome sequencing in normal brain and glioma tissues [15]. However, the effect of circEXOC6 was not further validated. In agreement with the previous outcome, our study showed that circEXOC6 was overexpressed in glioma tissues and cells, and downregulated circEXOC6 evidently suppressed cell proliferation, migration, invasion, glycolysis, and angiogenesis of glioma cells, and boosted cell apoptosis.

In our study, the online website of circinteractome could predict that circEXOC6 has a complementary sequence with miR-433-3p, and dual-luciferase reporter assay and RIP assay results further ascertained their relationship. As a tumor suppressor factor, miR-433-3p was considered to play an important role in human multiple malignant cancers, such as hepatocellular carcinoma [29], esophageal squamous cell carcinoma [30], and NSCLC [31]. The data from this work showed that miR-433-3p expression was remarkably lower in glioma tissues and cells as compared with the corresponding control groups, which was similar to previous studies [32]. In addition, downregulated miR-433-3p could overturn the inhibition effect of circEXOC6 in the malignant progression of glioma cells. These results prompted that circEXOC6 might sponge miR-433-3p to monitor glioma cell progression.

The bioinformatics of starbase results suggested that miR-433-3p had binding sites with the 3′-UTR region of frizzled class receptor 6 (FZD6). FZD6, a member of the “frizzled” gene family, participates in the development of different human diseases, such as downregulated FZD6 repressed cell proliferative and migratory abilities in gastric cancer cells [33]. The expression level of FZD6 was strikingly elevated in colorectal cancer [34]. Similarly, the FZD6 expression level was obviously enhanced, and elevated FZD6 reversed miR-433-3p overexpression-suppressed cell progression of glioma, which was coincident with the previous research [35]. These data indicated that miR-433-3p regulated FZD6 to reveal the molecular mechanism of glioma cells. Nevertheless, the various downstream pathways of FZD6 are still elusive in glioma cells, which requires further studies in the future.

As indicated previously, our studies manifested that circEXOC6 regulated the FZD6 expression through sponging miR-433-3p. Knockdown of circEXOC6 repressed cell proliferation, migration, invasion, glycolysis, and angiogenesis, as well as promoted cell apoptosis in glioma cells by sponging miR-433-3p and reducing the FZD6 expression level. Hence, the data provided a new direction in glioma treatment.

## Supplementary Material

Supplementary Figure sm
