# Supplementary material for: Knockdown of circEXOC6 inhibits cell progression and glycolysis by sponging miR-433-3p and mediating FZD6 in glioma
Source: Transl Neurosci. 2023 Aug 2;14(1):20220294. doi: 10.1515/tnsci-2022-0294 (PMC10404894; doi:10.1515/tnsci-2022-0294)
Supplement: Supplementary Figure sm [file tnsci-2022-0294-sm.pdf]

Supplementary material

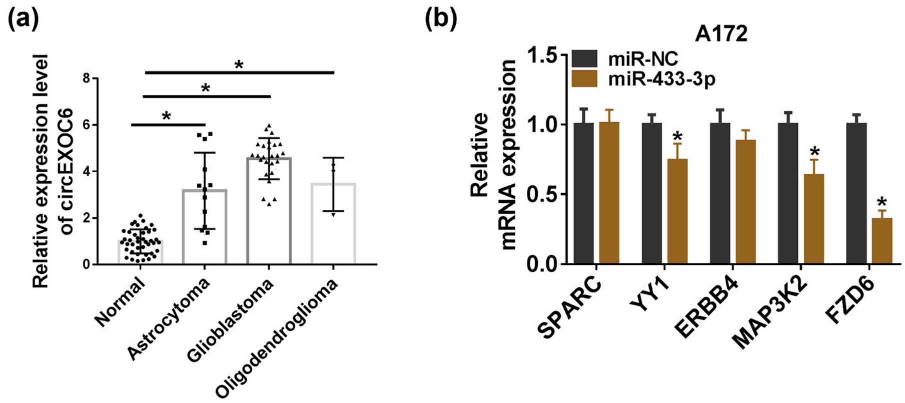

Figure S1: The expression of circEXOC6 and candidate mRNA expression. (a) CircEXOC6 expression in different types of glioma and normal tissues was detected by qRT-PCR. (b) The expression of candidate mRNA in A172 cells transfected with miR-NC/miR-433-3p mimic was measured by qRT-PCR. All experiments were performed in triplicate, with each independent experiment set 3 times to generate an average value. *$P < 0.05$.
